# Aptamer Renaissance for Neurochemical Biosensing

**DOI:** 10.1021/acsnano.3c09576

**Published:** 2024-01-18

**Authors:** Annina Stuber, Nako Nakatsuka

**Affiliations:** Laboratory for Biosensors and Bioelectronics, ETH Zürich, 8092 Zürich, Switzerland

## Abstract

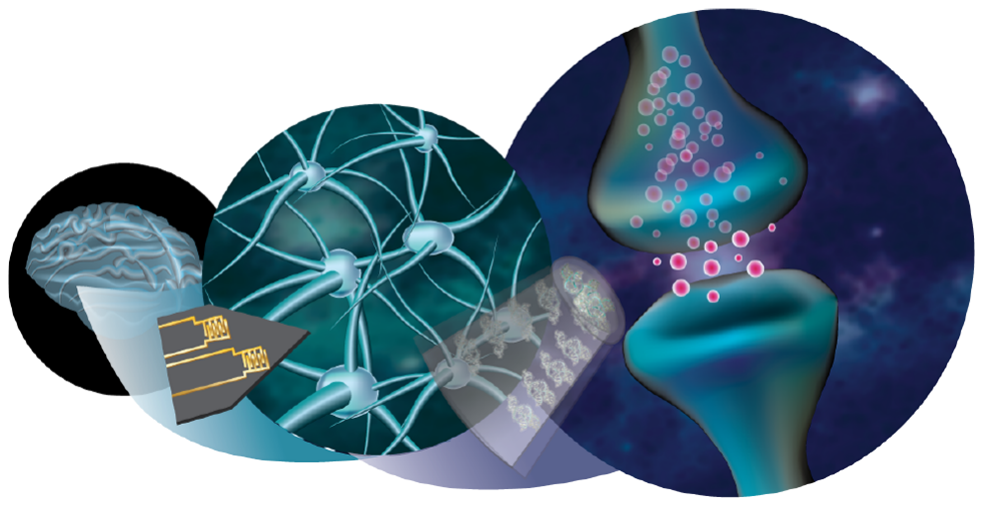

Unraveling the complexities of brain function, which is crucial for advancing human health, remains a grand challenge. This endeavor demands precise monitoring of small molecules such as neurotransmitters, the chemical messengers in the brain. In this Perspective, we explore the potential of aptamers, selective synthetic bioreceptors integrated into electronic affinity platforms to address limitations in neurochemical biosensing. We emphasize the importance of characterizing aptamer thermodynamics and target binding to realize functional biosensors in biological systems. We focus on two label-free affinity platforms spanning the micro- to nanoscale: field-effect transistors and nanopores. Integration of well-characterized structure-switching aptamers overcame nonspecific binding, a challenge that has hindered the translation of biosensors from the lab to the clinic. In a transformative era driven by neuroscience breakthroughs, technological innovations, and multidisciplinary collaborations, an aptamer renaissance holds the potential to bridge technological gaps and reshape the landscape of diagnostics and neuroscience.

## Introduction

Advances in human health necessitate the monitoring of distinct molecules within the complex milieu of biological fluids in the body. The continuous tracking of small molecules that orchestrate crucial biological processes offers insights into overall well-being. Additionally, the ability to trace disease biomarkers empowers early detection and facilitates personalized treatment approaches. In this context, biosensors have emerged as pivotal tools. Biosensors are specialized devices designed to react upon contact with specific molecules and subsequently convert this interaction into a measurable signal. In neuroscience, these tools are invaluable for monitoring small signaling molecules, such as neurotransmitters, that enable communication in the brain. Quantification of these chemical messengers has the potential to improve our limited understanding of fundamental brain function and unravel the complexities underlying brain disorders. While neurochemical biosensors hold great promise for applications in neuroscience, several technological gaps remain for practical implementation.

A significant bottleneck arises from nonspecific binding, a ubiquitous phenomenon in complex biological milieu where interfering molecules adhere to the sensor surface, leading to erroneous recordings, surface biofouling, and restricted sensitivity.^1^ This challenge highlights the need for high selectivity, particularly in distinguishing specific from nonspecific interactions, given the coexistence of structurally similar neurochemicals at comparable concentrations in the brain.^2^ Moreover, neurochemical biosensors should operate in a label-free manner to monitor endogenous molecules in their native environment with adequate spatial resolution while minimally affecting the system. Further, real-time monitoring with fast kinetics is essential to capture the dynamic brain processes. Biosensors must function within a sensitivity regime that enables the precise quantification of specific molecules across clinically relevant concentration ranges. Intricate neurochemical processes necessitate biosensors that are multiplexed to enable detection of multiple analytes simultaneously. Fulfilling these prerequisites, in concert with technological advancements and multidisciplinary collaboration, is crucial to realizing the full potential of biosensors in the field of neuroscience.

While the remaining challenges are highlighted, it is important to acknowledge the significant advancements in diverse neurochemical detection methodologies over recent years. Each of these approaches offers distinct advantages and limitations. For instance, magnetic resonance imaging (MRI) has enabled tracking of neurotransmitters such as dopamine at the whole-brain level in a minimally invasive manner.^3^ However, MRI requires labeling with contrast agents, and the high spatial coverage comes at the cost of resolution. Microdialysis coupled to analytical detection platforms offer multiplexed sensing capabilities, but remains an invasive technique with limited temporal resolution that impedes the capture of real-time neurochemical dynamics.^4,5^ Fast scan cyclic voltammetry (FSCV) offers millisecond-level temporal resolution and has made progress in addressing traditional challenges of molecular selectivity, but miniaturizing FSCV electrodes for improved spatial resolution sacrifices sensitivity.^6^ Genetically encoded optical sensors enable multiplexed detection, but concerns include system perturbations from sensor expression and the time-intensive development of sensors with physiologically relevant sensitivity for specific targets.^7,8^

In the realm of neurochemical biosensing, each technique occupies a distinct niche, and the prioritization of specific parameters often involves trade-offs that impact other measurement factors. Advances in fundamental neuroscience, striving for a comprehensive understanding of brain chemistry across diverse spatiotemporal resolutions and analyte concentrations, benefit from accessibility to a range of techniques. However, the clinical translation of neurochemical biosensors demands additional considerations. For instance, oral doses of levodopa, the dopamine precursor that can cross the blood–brain barrier, is the gold standard drug to treat patients suffering from involuntary motor control in Parkinson's disease. However, levodopa dosages are not personalized, despite patient-to-patient variations due to genetic differences or other influences such as diet and exercise. Dopamine metabolism is complex: toxicity can arise from high brain levodopa or high blood dopamine and patients experience severe side effects and encounter challenges with managing symptoms.^9^ Thus, monitoring dopamine levels at regular time intervals or, ideally, in real time in patient blood samples may offer personalized feedback for drug dosage. For such measurements, challenges arise due to the low concentrations of blood dopamine (as low as picomolar levels)^10,11^ and its susceptibility to oxidation,^12,13^ causing degradation. Thus, personalized treatments for patients with brain disorders such as Parkinson’s disease require accessible sensors that are selective, sensitive, and capable of fast, direct neurochemical quantification in biofluids.

**Figure 1:**
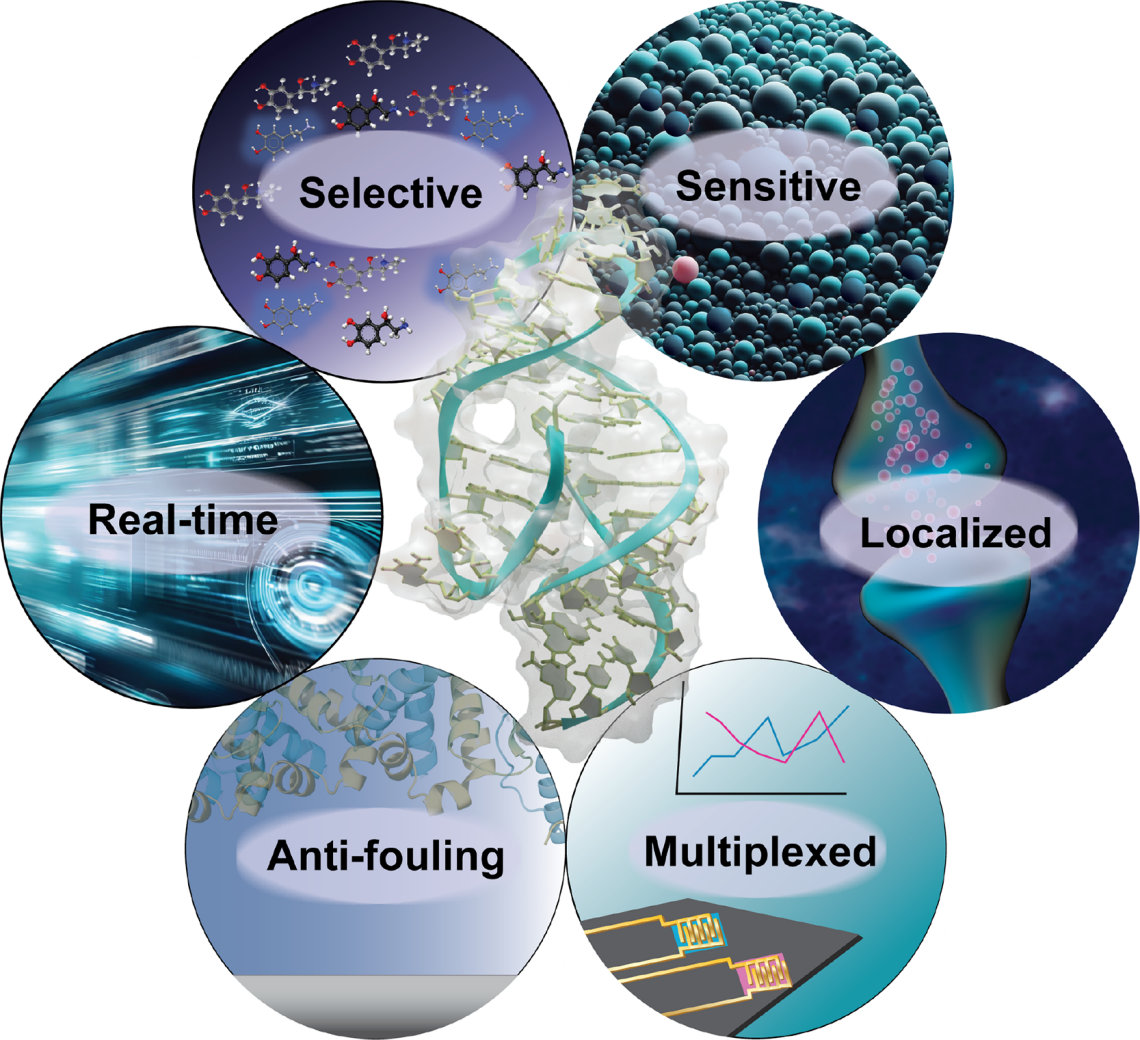
Graphical representation of the important characteristics for next-generation aptamer-based neurochemical biosensors. Certain images were created with the assistance of DALL-E 3.

This specific application of dopamine sensing in patient blood underscores the need for alternative technologies that combine high temporal resolution, sensitivity, and selectivity in biofluids. Aptamers are single-stranded oligonucleotides designed *in vitro* to bind specific analytes with high molecular selectivity in a reversible manner with tunable binding kinetics. These properties have led to aptamers emerging as advantageous bioreceptors for next-generation biosensors. Aptamers can be isolated for diverse targets with high throughput to address the expansive range of small molecules in the brain beyond neurotransmitters. Integration of aptamers into label-free affinity platforms with electronic readout enables sensitive, label-free, real-time, and localized measurements with potential for clinical translation (Figure 1). A resurgence of interest in aptamers as molecular recognition tools could bridge technological gaps and reshape diagnostics and neuroscience research. However, unlocking the full potential of aptamer-based biosensors (aptasensors) requires careful considerations, which we will highlight in this Perspective.

## Aptamers as Molecular Recognition Elements

Nucleic-acid aptamers have received significant attention as versatile affinity reagents since their discovery over three decades ago.^14,15^ In contrast to traditional protein-based receptors such as antibodies, aptamers offer enhanced stability, cost-effective production, minimal batch-to-batch variation, and facile chemical modification for integration into sensing platforms.^16^ The modular nature of aptamer isolation enables the high-throughput discovery of selective sequences with tunable parameters. Aptamer selection employs an iterative process termed the systematic evolution of ligands by exponential enrichment (SELEX), where a large library of oligonucleotides is screened to identify candidates with optimal binding affinities for the target of interest. Aptamers can be designed to differentiate structurally similar molecules via counter-SELEX, where candidate sequences are exposed to nontarget molecules and those that interact are eliminated.^17^ Recent advances in SELEX technologies have significantly improved the quality of isolated aptamers.^18,19^

Despite the numerous advantages associated with aptamers, only a limited number of sequences have been translated for human health. It is important to emphasize that achieving analyte specificity alone is insufficient to develop a clinically viable aptasensor. In the following sections, we discuss important aspects to realize an aptamer renaissance.

### Designing and Tuning Aptamer Properties for Specific Applications

Designing aptamers with adequate affinities for certain small molecules has proven difficult.^20^ However, the analysis of how individual functional groups influence aptamer-target recognition has facilitated structure-guided aptamer selections for previously elusive targets.^21^ Importantly, this insight into functional-group-guided aptamer selections holds the potential to enhance training sets for future computational *in silico* design of aptamers.

Once aptamers have been designed, their efficacy relies on the comprehensive characterization of their thermodynamic properties for molecular recognition, including their binding affinities (*K*_D_) and kinetic rates. These parameters are pivotal in assessing aptamer suitability for specific applications. In biosensing, the linear detection range where concentration-specific signal changes are quantifiable, must correspond to the sensing window relevant to different diagnostic needs. Optimizing aptamer *K*_D_ values is not only critical for accurate molecule quantification but also significantly impacts biosensor response times. The *K*_D_ value is defined as the ratio of the dissociation rate (*k*_off_) to the association rate (*k*_on_). Thus, high-affinity recognition molecules, characterized by a low *K*_D_ (indicating that the association rate dominates over the dissociation rate), are favored to achieve low detection limits. Alternatively, low-affinity bioreceptors with a high *K*_D_ (implying the dissociation rate exceeds the association rate) exhibit fast binding kinetics, making them suitable for real-time monitoring applications. Response times with subsecond temporal resolution are especially important for tracking neuronal signaling in the brain.

Recent technological advances have enabled the optimization of aptamer affinities by streamlining the SELEX process. The N2A2 was developed by modifying the commercialized *illumina* DNA sequencing platform and introduces specific base mutations into an aptamer library.^22^ Subsequently, N2A2 conducts high-throughput identification of variations that enhance or inhibit the binding affinity to the target. This comprehensive mapping approach identifies key mutations that optimize aptamer binding properties, thereby significantly expediting the aptamer development process.^23^ Alternatively, the “Pro-SELEX” method leverages microfluidic technology to profile the binding performance of individual aptamer candidates at varying target concentrations within a single selection round.^24^ This approach results in the generation of aptamers with programmable binding affinities.

In addition to implementing affinity tuning within the selection process, post-SELEX modifications can engineer the aptamer thermodynamic properties. To extend, narrow, or tune the dynamic sensing range, mechanisms such as cooperativity, allostery, and sequestration have been harnessed, as well as the introduction of specific mutations into the original aptamer sequence.^25,26^ Aptamers can be engineered such that elongating or shortening certain nontarget-binding regions can tune the affinity as well as the binding kinetics to the desired sensing range.^27,28^

### Characterizing Aptamer Binding in Relevant Environments

Considering the importance of aptamer thermodynamics in specific applications, it is crucial to characterize the binding properties of individual sequences thoroughly. Despite their initial promise, aptamers have faced implementation challenges in biological systems, often stemming from discrepancies between the conditions used during aptamer selection and the actual deployment environments. During the SELEX process, parameters, such as the pH, temperature, and ionic content, should resemble the final application environment. Such selection conditions are of paramount importance as minor variations can induce alternative oligonucleotide secondary structures, resulting in modified binding pockets incapable of target recognition. Therefore, it is imperative to investigate aptamer-target binding in solutions that closely mimic the relevant biological environment.^29^

In addition to conducting SELEX under relevant environmental conditions, an equally critical aspect involves characterizing aptamer-target binding in specific solutions using various complementary methods. Inadequate aptamer characterization for reported sequences prior to integration into biosensing platforms is a significant factor that contributes to failures in translating aptasensors. Several methodologies are available for characterizing aptamer-small-molecule interactions in solution, including isothermal titration calorimetry, fluorescence-based assays, or microscale thermophoresis.^20^ Alternatively, to replicate the behavior of aptamers immobilized on the surface of affinity platforms, techniques such as surface plasmon resonance or biolayer interferometry can be used to determine on-chip binding affinities and kinetics *in situ*.^30^

### Overcoming and Exploiting the Debye Length Limitation via Structure-Switching Aptamers

**Figure 2:**
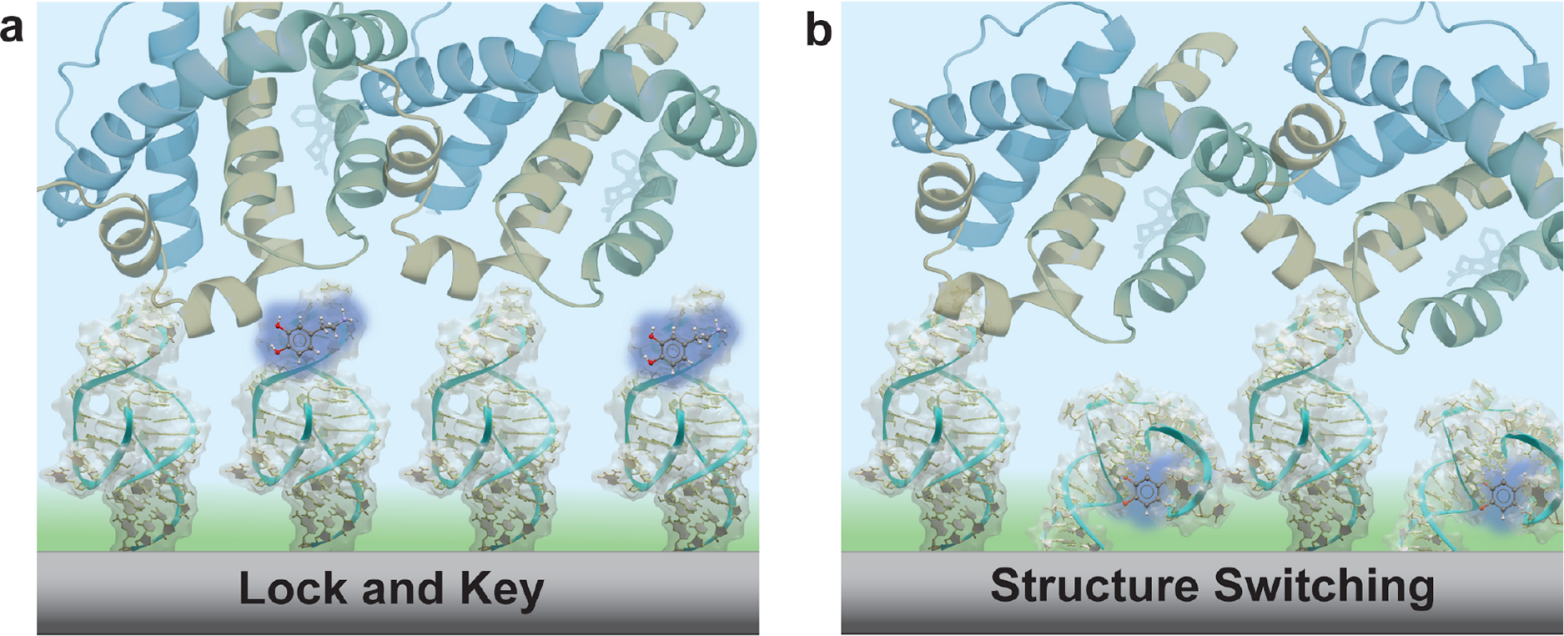
Schematic of aptamers binding to their small-molecule targets in biological environments. (a) Lock and key binding of targets to aptamers occurs outside the Debye length (represented by green shading). Thus, differentiation of specific vs nonspecific binding to the aptamers is challenging. (b) Structure-switching aptamers undergo conformational changes upon target binding, altering the negative charge density within or near the Debye length. Despite the inevitable nonspecific binding, the amplified signal of the aptamer rearrangement with associated counterions is transduced close to the sensor surface in the sensitive regime.

However, characterizing aptamer-target binding, while essential, represents just one facet of aptasensing platforms. It is equally important to consider the signal transduction mechanism, which translates the binding event into an electrical signal. Electronic biosensors offer distinct advantages in neurochemical sensing, where stringent requirements for sensitivity, real-time monitoring, miniaturization, and multiplexing are paramount (Figure 1). However, for electronic biosensors to function effectively in biological environments, a fundamental limitation defined as the Debye length (λ_D_) must be overcome.^31^ This parameter defines the limited range within which the electronic sensor surface remains sensitive to charge modulation. The λ_D_ is influenced by the ionic content of the environment and is restricted to <1 nm in physiological conditions; beyond this distance from the surface, an exponential decrease in sensitivity is observed due to ionic screening. The conventional approach of employing “lock and key” aptamer capture mechanisms, where the small molecule binds to a preformed aptamer binding pocket outside of λ_D_, often cannot be distinguished from nonspecific interactions (Figure 2a). This issue becomes particularly pronounced when detecting small molecules with limited mass and charge. Discriminating between specific vs nonspecific binding is difficult, given the propensity of the negatively charged oligonucleotide backbone to attract electrostatic interactions.

Structure-switching aptamers provide a solution to overcome the limitations of λ_D_ while amplifying small-molecule binding effects.^31−33^ These aptamers undergo significant conformational rearrangements upon target capture, and the signal is amplified by the movement of counterions associated with the negatively charged oligonucleotide backbone. These aptamers facilitate signal transduction in close proximity to the sensing surface through target-induced structural rearrangements that alters the charge density within the sensitive region (Figure 2b). This mechanism harnesses the λ_D_ to an advantage, allowing nonspecific binding events to occur at distances >1 nm where the sensing area is passivated by assembled aptamers. Consequently, only target-induced negative charge rearrangements are observed as the electronic signal, ensuring target specificity of the biosensor.

It is important to note that the mentioned advantages are specifically relevant to small-molecule sensing by using structure-switching aptamers. When larger target molecules such as proteins with significant charge and mass are detected, the need to amplify the binding event for signal transduction becomes less crucial. Sensing of larger analytes is primarily hindered by nonspecific binding. Nevertheless, specific biosensor designs including diffractometric, molecular, and stochastic approaches, have proven effective in mitigating this challenge.^1^

### Characterizing the Structure-Switching Dynamics of Aptamers

In the context of developing aptasensors for neurotransmitter detection within the brain milieu, the role of structure-switching aptamers becomes pivotal. These aptamers can be systematically selected using advanced SELEX procedures.^17,19,34^ As emphasized prior, characterization of aptamer-target interactions in specific environments is of critical importance, not just for aptamer-target binding but also for structure-switching aptamers. A DNA aptamer targeting the neurotransmitter dopamine illustrates the profound influence of ionic species on aptamer conformational dynamics. Notably, this dopamine-specific aptamer was isolated in solutions mimicking the ionic content in the brain (artificial cerebrospinal fluid, aCSF), where divalent cations such as magnesium and calcium are present.^31^ In aCSF, the dopamine aptamer exhibited large conformational rearrangements upon target recognition.^35^ In contrast, in solutions typically used for *in vitro* biosensor validation lacking divalent cations (phosphate buffered saline, PBS), the dopamine aptamer structure switching was severely hindered.^36^ Consequently, when these dopamine aptamers were integrated into biosensors that transduce aptamer rearrangement as a measurable electronic signal, the magnitude of the sensor response was significantly amplified in aCSF vs PBS.^36^

Investigating aptamer conformational dynamics is crucial for understanding their influence on the detection mechanism when integrated into biosensing platforms. Various strategies enable tracking of the aptamer structure switching in solution. For example, fluorescence-based methods (e.g., Förster resonance energy transfer) monitor the distance between fluorescence reporters attached to specific locations on the oligonucleotide backbone.^37^ Such methods offer insights into the movement of specific aptamer motifs within nanoscale distances. Alternatively, circular dichroism spectroscopy provides a more global perspective on changes in aptamer secondary structures upon target recognition.^38^ Nuclear magnetic resonance spectroscopy can elucidate tertiary aptamer-target complex structures, although this method is most effective at low salt conditions, which may not mimic the optimal aptamer environment.^39^ An alternative method to determine aptamer 3-D structures is X-ray crystallography, however, the process of obtaining aptamer crystals is challenging and laborious, and the crystal structure may differ from the solution-specific conformation.^40^

When aptamers are immobilized on affinity platforms, the magnitude of structure switching may differ due to the reduced degree of conformational freedom.^41^ Quartz crystal microbalance with dissipation monitoring (QCM-D) monitors the behavior of surface-tethered aptamers by tracking the mass assembly on chip surfaces. Small-molecule binding is observable through changes in solution ion adsorption to hydrophilic aptamers based on their conformation.^42^ Thus, QCM-D recordings can indicate either the expulsion or adsorption of solution ions as the aptamer layer compresses or expands, respectively.^43,44^ While QCM-D observes ensemble changes of the aptamer monolayer, interactions at the single-molecule level can be studied using computational models such as molecular dynamics (MD) simulations.^44^ These simulations can restrict the 5′ end of the aptamer functionalized to the sensor surface to mimic immobilization. Conducting multiple complementary techniques can lead to a comprehensive mechanistic understanding of how aptamer structure switching influences biosensor performance.

### Harnessing Structure-Switching Aptamers Using Field-Effect Transistors

**Figure 3:**
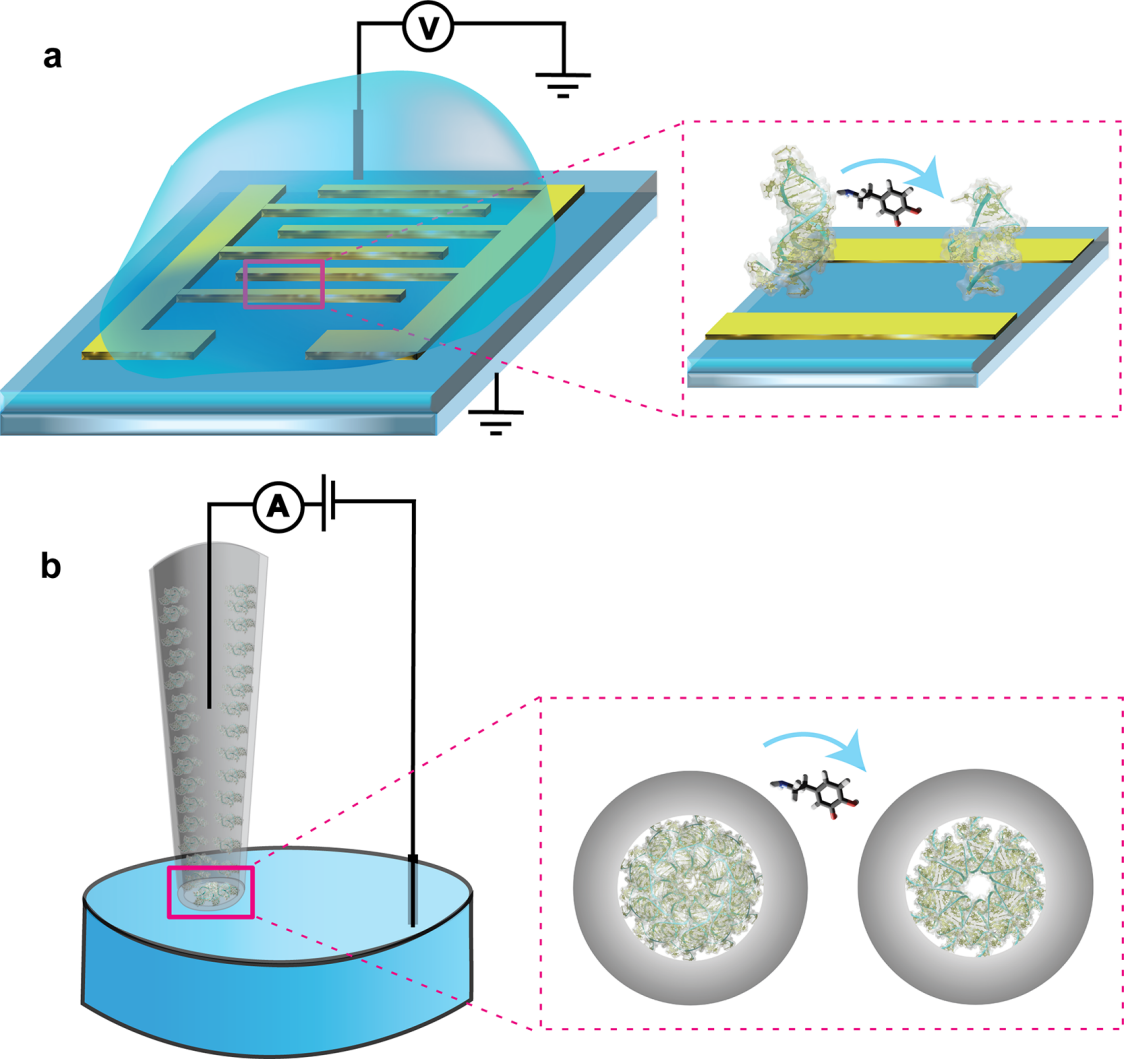
Schematics of affinity platforms that transduce aptamer structure-switching as measurable electronic signals. (a) Aptamer-modified field-effect transistors comprise a semiconducting channel (blue surface) that connects the metallic source and drain electrodes (gold). Measurements are conducted in solution with a solution reference electrode. The zoom shows aptamers immobilized on the semiconducting channel that undergo structure switching upon target recognition. The charge rearrangement is transduced as a change in electric current through the semiconducting channel. (b) Aptamer-functionalized nanopipettes measure ionic flux through the nanopore at the tip by applying a voltage between two quasi-reference counter electrodes: one inside the nanopipette and one in the measurement solution. The zoom represents a view into the orifice of the nanopipette occluded with aptamers. Upon target binding, the rearrangement of the negatively charged aptamer backbone induces a change in charge distribution within the nanopore. This alteration in charge density influences the ionic flux measured through the nanopore.

Charge-sensitive affinity platforms such as field-effect transistors (FETs) are crucial for harnessing the characteristic properties of structure-switching aptamers. Conventional FETs typically employ semiconducting channel materials that are either *n*- or *p*-doped that connect metallic source and drain electrodes to monitor variations in electric fields above the channel surface (Figure 3a). In essence, alterations in surface electric fields within λ_D_ are manifested as changes in resistance to electron flow within the semiconductor. The FET channels can be functionalized with aptamers using surface chemistry procedures.^45^ The binding of target molecules to the aptamers effectively gates the FET channels by modulating surface potentials, which control charge carrier populations within the channels. These alterations in transconductance contribute to a significant amplification of the detection signal for small-molecule targets. Notably, FET channels constructed from nanometer-thin films,^31,46^ or low-dimensional materials,^47−49^ offer sensitivity enhancement due to changes in the surface potential penetrating the entire semiconducting material.

Aptamer-functionalized FETs detected small-molecule neurotransmitters (serotonin and dopamine) as well as neutral (glucose) and zwitterionic (sphingosine-1-phosphate) molecules.^31^ These aptasensors demonstrated low detection limits in the femtomolar range and exhibited high selectivity, even when immersed in undiluted biological environments. Importantly, the aptamers employed in this study were initially isolated under conditions designed to mimic the ionic milieu of specific biological systems. Further, these aptamers were extensively characterized both before and after integration onto nanoscale (∼4 nm) indium oxide FET channels.^46^ Subsequently, aptamer-FETs were miniaturized for implantable neural probes, with widths ranging from 50 to 150 μm.^50^ These probes enabled the real-time detection of stimulated serotonin release in awake mice *in vivo*. The successful translation of this technology from *in vitro* experimentation to *in vivo* application can be attributed to systematic investigations of basic mechanisms that underlie aptamer-FET biosensing.

Although aptamer-functionalized FETs have demonstrated *in vivo* biosensing potential, ensuring biocompatibility, biostability, and reproducibility for long-term recordings (days to weeks) is critical. Three important aspects should be considered for continuous *in vivo* monitoring, especially in the context of neurochemical biosensing. First, we must consider the influence of implanted devices on local tissue responses. The physical insertion of a probe into brain tissue causes local penetration injury, which initiates a cascade of inflammatory responses.^51^ The inflammation subsequently alters the sensing microenvironment, leading to sensor inaccuracy, instability, and often failure. A potential avenue to mitigate this issue involves the size reduction of implanted devices to minimize tissue strain and damage. Alternatively, flexible, stretchable, tissue-mimicking devices have shown promise in reducing chronic inflammation.^52,53^ Further, progress has been made in the area of “transient” electronics featuring biodegradable power sources and wireless data collection capabilities.^54^ Engineering such characteristics into aptamer-FET sensors has the potential to improve prospects for long-term monitoring and human use.

The second critical aspect for *in vivo* biosensing is biofouling, where nonspecific biological molecules adsorb onto sensing areas, leading to compromised sensor performance or failure.^1^ Planar biosensors with exposed recognition elements exhibit baseline drifts during prolonged measurements, especially in biological systems. The third concern pertains to the vulnerability of aptamers to the environment. Nucleases, natural enzymes in the body, degrade oligonucleotides by cleaving phosphodiester bonds, potentially leading to aptamer degradation.^55^ An approach to tackle this issue, involves the use of unnatural left-helix aptamers termed spiegelmers with artificial base orientations, preventing nuclease attachment.^56^ However, such modifications come at the cost of complex design and fabrication, which hinder development. Alternatively, aptamer stability can be enhanced by fortifying the bases using sugars or phosphate groups, reducing the likelihood of cleavage.^57^ Such substitutions, strategically positioned to increase nuclease resistance, have been implemented into SELEX procedures that are conducted within whole organisms to bolster the translation potential of aptamers.^58^

In the following section, we delve into an alternative nanoscale affinity platform designed to address the aforementioned challenges associated with continuous monitoring in biological systems. The miniaturization of the sensor to nanoscale dimensions serves to reduce tissue damage upon implantation. Further, the strategic confinement of aptamers within nanoscale orifices protects the bioreceptors from nuclease degradation while simultaneously circumventing biofouling by occluding nonspecific interferents.

### Confining Structure-Switching Aptamers Inside Nanopores

Quartz nanopipettes represent a solid-state platform ideally suited for facilitating the transduction of the charge rearrangement of structure-switching aptamers. Nanopipettes have the advantage of facile fabrication through laser pulling, resulting in pore openings ranging from microns to nanometers at the tip, contingent on specific pulling parameters.^59^ Additionally, quartz is chemically inert while being amenable to surface chemistry for aptamer integration. For measurements, nanopipettes are filled with the same solution as the immersion solution, and the ionic current is measured between two quasi-reference counter electrodes, one placed inside the nanopipette and the other in the bulk solution (Figure 3b). Applying a linear voltage between these two electrodes leads to a nonlinear measured ionic current due to asymmetric ion transport. This phenomenon manifests as the ion current rectification (ICR) effect, which is influenced by a combination of factors including the conical geometry of the nanopipette, the size of the nanoscale tip, and the surface charge distribution within the nanopore. When sensor geometries are kept constant, alterations in surface charge within the nanopore can modulate the measured current.

Given the sensitivity of the ICR effect to variations in surface charge, the motivation for integrating structure-switching aptamers inside of nanopipettes become evident. The aptamers modulate the ionic current through the nanopore in response to the presence of the target of interest. Serotonin aptamer-modified nanopipettes showed target specificity with a sensitive detection window spanning the picomolar-nanomolar range.^43^ Further, these serotonin sensors exhibited minimal responses to structurally similar molecules, validating their selective behavior. This sensor outperformed gold standard antibody-based methods such as enzyme-linked immunosorbent assay when quantifying serotonin release from human stem cell-derived serotonergic neurons.^60^ Notably, these recordings were conducted without sample pretreatment or dilution, offering label-free detection directly in biological media.^43^

**Figure 4:**
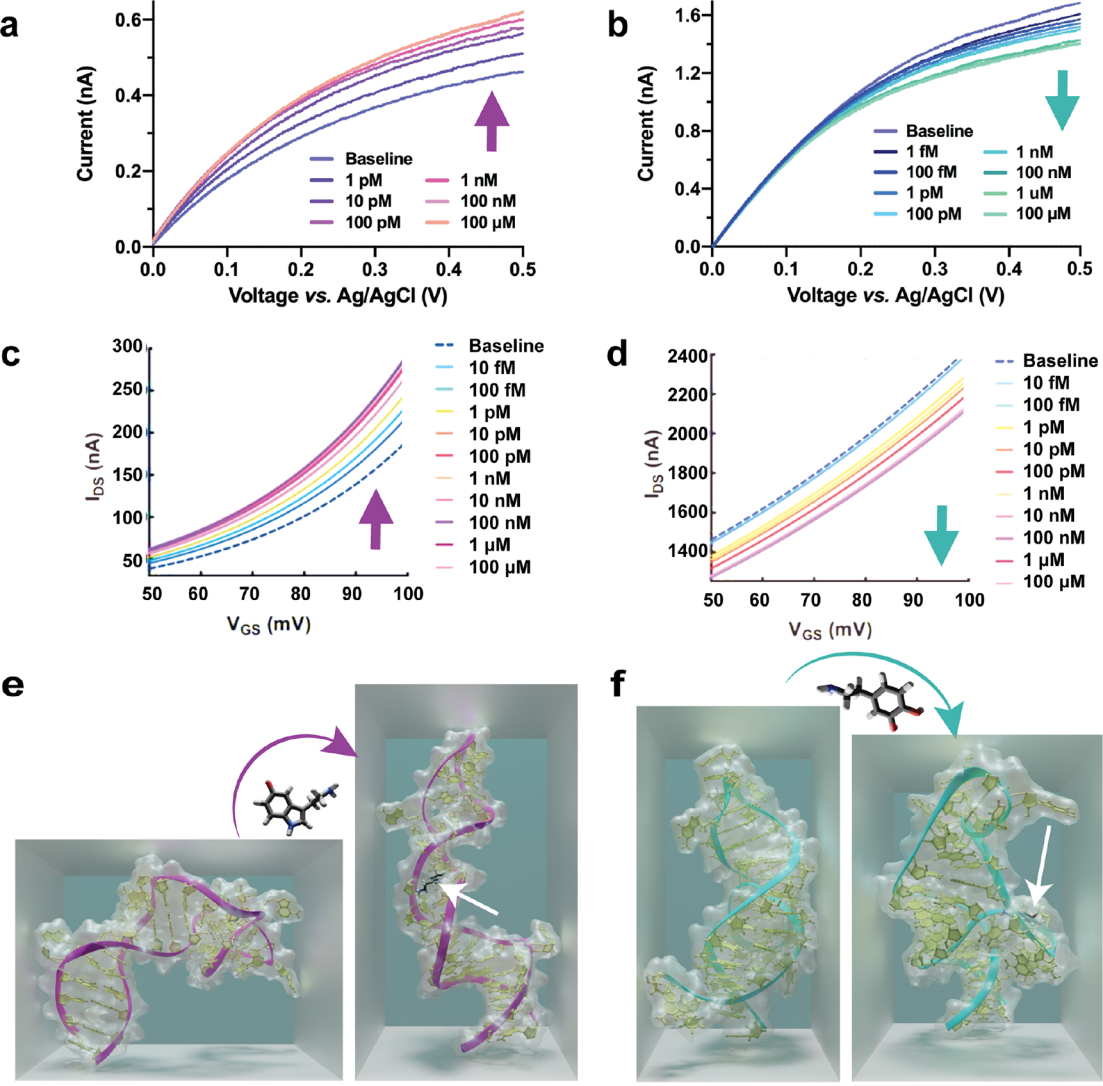
Correlating aptamer-modified sensing behavior to sequence-specific conformational dynamics. Field-effect transistors functionalized with (a) serotonin aptamers or (b) dopamine aptamers showed opposite directions of current change upon exposure to increasing concentrations of target analyte. Nanopipettes modified with (c) serotonin aptamers or (d) dopamine aptamers similarly showed divergent responses in measured current with increasing amounts of respective analytes. Molecular dynamics simulation of the (e) serotonin aptamer and (f) dopamine aptamer in the absence and presence of the target analyte revealed an elongation of the serotonin aptamer and a compression of the dopamine aptamer. White arrows indicate the analyte binding pocket. Divergent structure-switching behavior explains the opposite trends in biosensor behavior between serotonin and dopamine aptamers. Panel a adapted with permission from ref (43). Copyright 2021 American Chemical Society. Panels c and d adapted with permission from ref (31). Copyright 2018 American Association for the Advancement of Science. Panels b, e, f adapted with permission from ref (44). Copyright 2023 American Chemical Society.

In a subsequent study, a dopamine aptamer-specific nanopipette sensor was developed, capable of detecting dopamine selectively within complex biofluids including human serum.^44^ A divergence in sensing behavior emerged between dopamine and serotonin sensors. When exposed to respective targets, serotonin sensors consistently exhibited an *increase* in signal response (Figure 4a)^43^ while dopamine sensors showed a *decrease* in response (Figure 4b).^44^ Despite serotonin and dopamine sharing similar molecular mass and charge profiles under physiological conditions, this sensing behavior was consistently observed, extending to FET measurements for serotonin (Figure 4c) and dopamine (Figure 4d) aptamer-modified surfaces.^31^ Recent insights from MD simulations enabled visualization of the most probable 3-D aptamer structure and the target-dependent conformational change.^44^ Inherent opposite directions of conformational change exhibited by serotonin aptamers (elongation, Figure 4e) vs dopamine aptamers (compression, Figure 4f), resulted in contrasting signal observation. These findings underscore the importance of in-depth aptamer characterization and mechanistic investigations in the development of functional aptasensors.

In addition to serving as highly specific, selective, and sensitive label-free neurochemical biosensors, aptamer-modified nanopipettes offer distinct advantages specifically for the field of neuroscience. Neurotransmitters are released in the synapse that spans 20–50 nm between two neurons for neuronal communication. Consequently, synaptic measurements inherently demand sensors that approach these nanoscale dimensions. While carbon-fiber nanoelectrodes with increasingly smaller dimensions have been developed for electrochemical measurements in proximity to synapses, it is important to note that the sensor sensitivity scales with the electrode area.^61^ Moreover, these nanoelectrodes expose their sensing surfaces to the surrounding environment, rendering them susceptible to biofouling. In contrast, aptamer-modified nanopipettes offer an effective solution by confining the sensing area.

Further, nanopipettes offer seamless translation for neuroscience applications *in vitro* and *ex vivo* due to their compatibility with patch clamp setups. The miniaturized tip size of nanopipettes not only minimizes tissue damage upon implantation but also holds potential for intracellular measurements within individual neurons in brain slices.^62^ While the nanoscale tip size of nanopipettes offers advantages in minimizing tissue damage upon implantation, it is important to acknowledge that these platforms lack the engineered flexibility seen in certain FET-based biosensors designed to match the Young’s modulus of brain tissue.^50,63^ Therefore, prior to *in vivo* deployment, additional engineering is necessary for nanopipette sensors to overcome their inherent rigidity and mitigate potential tissue damage resulting from movement.

The sensors can also be integrated into scanning probe methods such as scanning ion conductance microscopy (SICM) that allows 3-D topographical mapping of living cells with nanometer resolution in their physiological environments.^64^ High-speed, time-resolved SICM enables visualization of nanoscale topography with a subsecond temporal resolution. While SICM can achieve subsecond scanning speeds, the temporal resolution of sensing within aptamer-functionalized nanopipettes depends on analyte concentration, potentially not aligning with this high-speed technology. Although aptamer capture and release of analytes can occur at comparable subsecond time scales, in nanoscale confinement, released analytes may get trapped and recaptured by neighboring aptamers within the orifice. This densely assembled mesh of aptamers allows real-time detection when conducted in environments such as acute brain slices where low amounts of neurochemicals are released and quickly reuptaken.^65^ However, higher concentrations result in sensor saturation and mandate a reset protocol to release the accumulated molecules.^44^ Thus, careful consideration of temporal dynamics and analyte concentration is crucial in studying specific biological processes with aptamer-modified nanopipettes.

A remaining challenge in nanopipette sensor technology is the throughput limitation, particularly when compared to platforms such as FETs that allow for cost-effective, wafer-scale production.^46,66^ Consequently, FET-based sensors offer a significantly increased number of readouts, facilitating more robust statistical analysis. A potential route for upscaling single nanopipette measurements involves the use of nanopore arrays. Recently, stalactite (conical) nanopores were designed and fabricated in an array of 900 pores in 400 μm^2^ with individual pores as small as 3 nm.^67^ These nanopore arrays may be modified with aptamers. If recordings from individual nanopores were feasible, then this platform would enable localized monitoring of chemical release over a wide field of view. Such a development could significantly expand the capabilities of aptamer-modified nanopores for diverse biological systems.

### Tackling Remaining Challenges of Aptasensing in Complex Milieu

Aptamer-functionalized FET sensors and nanopipettes have emerged as valuable tools for biosensing in biological environments due to their high sensitivity to changes in electric charge. While this charge sensitivity is advantageous for small-molecule detection, it comes with a caveat. These sensors are inherently sensitive to environmental fluctuations, including variations in temperature, pH, or ionic strength. An example of the challenge posed by environmental flux is observed in neural depolarization, a phenomenon accompanied by rapid and localized alterations in the ionic content. Additionally, tumorigenic tissues often exhibit altered pH levels when compared to their healthy counterparts.^68^ The dynamic nature of biological systems underscores the complexity of translating biosensors into clinical settings.

Conducting differential measurements using carefully designed control sensors can mitigate this inevitable challenge.^69^ Installing both a control and specific sensor under the same environmental conditions would account for nonspecific variations in the signal. In this context, an ideal control sensor would be functionalized with a scrambled sequence that preserves the same number and type of nucleotide as the aptamer but in an alternate order, effectively eliminating specific recognition to the target. Thus, this control sensor with similar properties such as receptor size and charge would experience comparable nonspecific binding while remaining inert to the analyte of interest. However, it is important to emphasize that having a control sensor in spatial proximity to the specific sensor does not replace the need for thorough characterization of aptamer binding under specific environmental conditions that may be encountered. For instance, a scrambled aptamer would not serve as a control for environmentally induced alterations in the binding pocket of the specific aptamer. The screening of diverse environmental conditions for the specific sequences deployed must precede differential measurements.

After specific aptamer binding is validated under specific conditions, it is then crucial that the specific and control sensors experience the same environmental conditions. To achieve the close proximity of two independent sensors, microfabrication techniques and selective functionalization methods become essential tools. Fortunately, FET-based platforms are compatible with conventional microfabrication processes and large-area arrays of hundreds of FET sensing regions can be generated to facilitate extensive multiplexing.^70^ Nevertheless, a central challenge in achieving differential biosensing lies in the effective integration of distinct recognition elements in specific locations.

Strategies for immobilizing two different sequences on transistors with adequate spatial separation (millimeter scales) include the use of polymer masks that protect sensor areas for independent functionalization^71^ or the use of microfluidics.^37^ Further, electrografting of different functional groups such as carboxylic acid and amine groups onto graphene-FETs has shown promise for the modification of two different aptamers separated by <100 μm.^72^ However, scaling such chemical methods to a broader array of different aptamers remains challenging. Such approaches enable not only self-referencing but also multiplexing, the detection of several different molecules simultaneously. In the realm of neuroscience where neurotransmission inherently involves the co-release of various release of neurotransmitters,^73^ the capacity for multiplexed detection holds particular significance.

Similarly, for aptamer-functionalized nanopipettes, measuring the specific sensor in parallel with a control sensor functionalized with a scrambled sequence can serve as an adequate reference. However, the distance between the two nanopipettes must be controlled to ensure relevant referencing. To perform measurements near synapses or intracellularly, the reference sensor would need to be positioned at nanoscale distances from the specific sensor to ensure that both nanopores observe the same environment. One approach to achieve this daunting requirement involves integrating the two sensors into a single capillary separated by a 20 nm-thick septum. These double-barrel nanopipettes with two parallel nanopores can be fabricated using laser pulling similarly to conventional nanopipettes.^74^ Beyond the double barrel, having multiple independent barrels adjacent to one another and separated by tens of nanometers,^75^ would enable multiplexing and referencing for nanopipette sensing. A remaining challenge lies in the selective functionalization of each barrel within nanoscale regions and the prevention of cross-contamination of oligonucleotides between adjacent nanopores.

While integrating multiple biorecognition elements into micro- to nanoscale distances enables highly localized probing, this miniaturized system can compromise the ability to obtain a comprehensive spatial overview. To address this limitation, one strategy is to increase the number of sensors deployed concurrently. However, the number of deployable sensors will be constrained by factors such as the dimensions and expenses associated with the external electronics. To obtain a comprehensive chemical-spatial overview, these electronic sensors could be integrated with genetically encoded fluorescent biosensors. Combining optical sensors that enable visualization of a large spatial distribution with aptamer-based probes that measure from localized regions will expand the field of neurochemical sensing.^76^ Variants of dopamine-specific fluorescent sensors have been developed, with typically nM *K*_d_ values.^8^ When these optical sensors are combined with aptamer-based electronic sensors with fM to nM detection ranges, the sensing window would also be significantly expanded.

Such integrated approaches offering both high spatial resolution and comprehensive chemical mapping hold immense potential for advancing the field of neurochemical sensing. An alternative route to achieve spatially resolved imaging of small-molecule secretion involves using hydrogel matrices embedded with fluorescent aptamers.^77^ Target-induced conformational changes of the fluorescent aptamers generate a measurable change in the fluorescent signal. Cells can be cultured on this aptamer-integrated hydrogel surface, and the secretion of molecules activates localized fluorescent aptamers, providing a real-time visualization of small-molecule release. However, this method is limited by microscopy resolution and is only suited for *in vitro* systems. Additionally, the presence of nucleases in the cell culture medium may lead to aptamer degradation over time.^78^ Despite remaining challenges, the integration of fluorescent spatial information with localized probes holds promise to advance our understanding of the intricate chemical landscape underlying neural communication.

## Conclusions and Outlook

The convergence of an aptamer renaissance with recent advances in neuroscience presents a transformative opportunity to enhance our understanding of fundamental brain (dys)function. Structure-switching aptamers, with their intrinsic selectivity and characteristic structural rearrangements upon target binding, have emerged as ideal recognition elements in chemical biosensors. However, we emphasize the importance of characterizing individual aptamer sequences within environments mimicking the final biological system to ensure robust translation. Our current toolbox for neurochemical sensing requires expansion to construct a comprehensive map of the chemical networks in the brain. Such insights would improve our understanding of psychiatric and neurodegenerative diseases such as Major Depression, Alzheimer’s disease, and Parkinson’s disease. Beyond the realm of neuroscience, strategies involving structure-switching aptamers are generalizable, rendering these technologies versatile for diverse applications in human health.

As we push the boundaries toward finer spatial resolution with increasingly smaller sensing platforms, we sacrifice a broader view of chemical flux. The integration of various neurochemical biosensors tailored for specific applications may address these remaining limitations effectively. We envision that aptasensors will not only serve as tools for fundamental research but will become of clinical importance enabling precise monitoring of trace-level small-molecule biomarkers in biofluids. Recent reports of aptamers capable of traversing the blood–brain barrier^79^ imply promising avenues for these biomolecules, not only in the context of aptasensors but also as potential therapeutic agents. Just this year, a human whole-body dynamic pharmacokinetics study was conducted using radiolabeled aptamers to evaluate their biosafety and circulation characteristics.^80^

**Figure 5:**
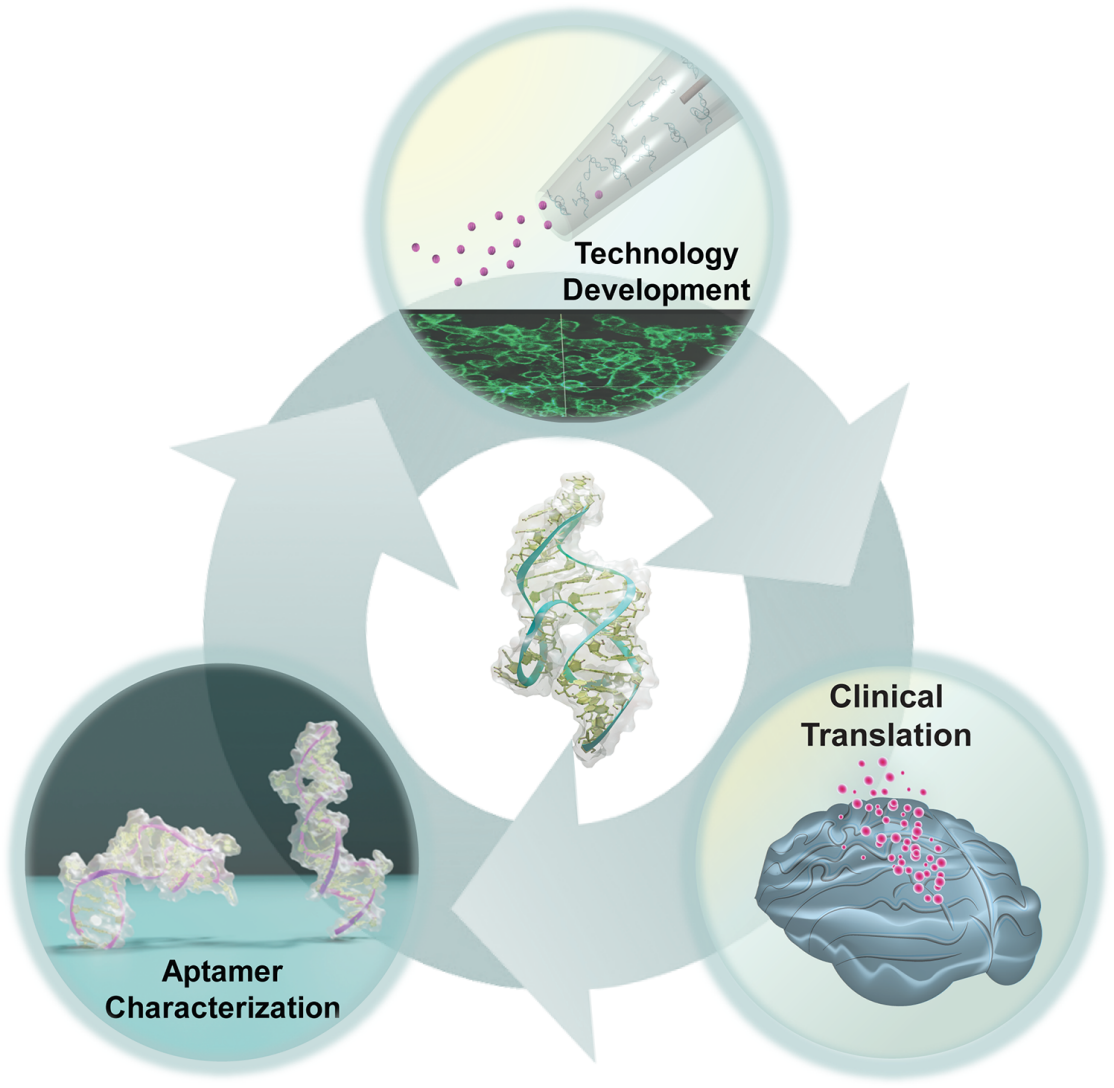
Iterative development of aptamer-based biosensors (aptasensors) relies on thorough characterization and structural understanding of aptamer-target interactions. This knowledge drives technological development of aptasensors that function in complex biological environments, facilitating clinical translation. As aptasensors monitor small molecules to advance our understanding of health and disease, new questions arise, necessitating the design of next-generation biosensors that require comprehensive characterization, creating a cyclic process.

Looking ahead, aptamers, which can function as both diagnostic and therapeutic agents, could facilitate closed-loop systems. In these systems, the detection of specific biomarkers could trigger targeted drug release, holding promise for personalized medicine. The design of such systems for clinical translation necessitates a foundational understanding of the fundamental mechanisms governing aptamer-target interactions, rendering this process cyclical (Figure 5). It is evident that multidisciplinary approaches at the intersection of chemistry, engineering, and neuroscience play pivotal roles in translating technologies from the lab to the clinic.
