# Correction to “Aptamer Renaissance for Neurochemical Biosensing”

**DOI:** 10.1021/acsnano.4c04993

**Published:** 2024-05-07

**Authors:** Annina Stuber, Nako Nakatsuka

**Affiliations:** Laboratory for Biosensors and Bioelectronics, ETH Zürich, 8092 Zürich, Switzerland

In the Figure 4 caption, the labels were switched for panels (a, b) and (c, d), where the data from (a) and (b) are from nanopipettes while (c) and (d) are from field-effect transistors.

Therefore, the text should read as follows:

Correlating aptamer-modified sensing behavior with sequence-specific conformational dynamics. Nanopipettes modified with (a) serotonin aptamers or (b) dopamine aptamers showed opposite directions of current change upon exposure to increasing concentrations of target analyte. Field-effect transistors functionalized with (c) serotonin aptamers or (d) dopamine aptamers similarly showed divergent responses in measured current with increasing amounts of respective analytes.

This correction does not affect the results or conclusions of the paper.

